# Prenatal Diagnosis of Cystic Fibrosis and Hemophilia: Incidental Findings and Weak Points

**DOI:** 10.3390/diagnostics10010007

**Published:** 2019-12-21

**Authors:** Marika Comegna, Giuseppe Maria Maruotti, Laura Sarno, Gustavo Cernera, Monica Gelzo, Maurizio Guida, Fulvio Zullo, Federica Zarrilli, Giuseppe Castaldo

**Affiliations:** 1 Department of Molecular Medicine and Medical Biotechnology, University of Naples Federico II, Via S. Pansini 5, 80131 Naples, Italy; marika.comegna@unina.it (M.C.); cernera@ceinge.unina.it (G.C.); monica.gelzo@unina.it (M.G.); giuseppe.castaldo@unina.it (G.C.); 2 CEINGE-Advanced Biotechnology, Via G. Salvatore 486, 80145 Naples, Italy; 3 Department of Neuroscience, Reproductive and Odontostomatological Sciences, University of Naples Federico II, Via S. Pansini 5, 80131 Naples, Italylaurettasarno@gmail.com (L.S.); maurizio.guida@unina.it (M.G.);

**Keywords:** prenatal diagnosis, hemophilia, cystic fibrosis, misattributed paternity, de novo mutation, complex alleles

## Abstract

Because of the progression of genetics and genomics, the demand for prenatal diagnosis (PD) for inherited genetic diseases has increased. However, several incidental findings may emerge during PD, like misattributed paternity, the evidence of disease in a parent, and the possible misinterpretation of the results because of complex alleles or de novo mutations that have several implications. In a retrospective observational study on all the couples referred to our Medical School (1993–2018) for PD of genetic inherited diseases (*n* = 1502), we selected the cases of PD for cystic fibrosis (CF, *n* = 239) and hemophilia A and B (HA, HB, *n* = 47), revising all incidental findings previously mentioned. We found one case in which a technical error led to PD of carrier in two siblings that were born affected by CF, four cases of misattributed paternity, eight cases of asymptomatic parents revealed as affected by CF transmembrane regulator (CFTR)-related disorders, a case of a novel complex allele that could have caused the diagnosis of CF in a carrier fetus, and a case of a de novo mutation in a mother (already a carrier) that caused hemophilia in a child that PD had revealed as healthy. We present these conditions as clinical cases and discuss the technical, clinical, ethical, and legal aspects to be considered.

## 1. Introduction

The demand for prenatal diagnosis (PD) for inherited genetic diseases is dramatically increasing because the number of known disease genes is growing and because the procedures for PD are becoming more effective. Non-invasive PD can be used to determine fetal sex, aneuploidies, and micro-deletions, but it can be applied to PD for inherited genetic diseases only in autosomal recessive diseases when parental mutations are different [1]. Thus, most PD for inherited genetic diseases is performed testing DNA from amniocytes sampled between the 15th and the 20th week of gestation or from chorionic villi (CV) sampled during the first trimester (10th–12th week).

In our Centre, that is the reference laboratory for molecular diagnostics of genetic diseases in Campania Region (Southern Italy, about 5 million inhabitants), we offered about 1200 PD for 73 different inherited diseases in 2013 [2], and now the figure has increased to about 1500 PD for 120 different diseases. We tested DNA from CV in more than 85% of cases. Among the requests for PD of inherited genetic diseases, one of the most frequent is cystic fibrosis (CF) [3], an autosomal recessive disease due to about 2000 different mutations in the gene encoding the CF transmembrane regulator (CFTR) protein. The disease, one of the most frequent genetic disorders in Caucasians (1:2500 newborn), typically appears with a severe phenotype that includes pancreatic insufficiency and early pulmonary inflammation also triggered by bacterial colonization that leads to respiratory insufficiency, even if a large heterogeneity of CF phenotype has been described [4].

Hemophilia A (HA, incidence: 1:5000 in newborn males) and hemophilia B (HB, incidence: 1:30,000 in newborn males) are the most frequent inherited bleeding diseases worldwide. Such disorders are X-linked and are due to the reduced activity of FVIII or FIX procoagulant factor, respectively. Two-thirds of HA or HB cases display a severe hemorrhagic phenotype and are associated with a residual activity of FVIII or FIX <1%. *Factor 8* (*F8C*) is the gene responsible for HA [5]. About half of severe cases are due to intron (IVS22) inversion, 5% of cases depend on IVS1 inversion, and the remaining 45% of cases are caused by thousands of different mutations also due to the high frequency of de novo mutations appearing on the X chromosome [5,6]. Hemophilia B, in turn, may be due to a myriad of different mutations spreading within the whole *Factor 9* gene (*F9*) [7]. Because of the severe phenotype of HA and HB, the demand for PD from couples with family history of the diseases is increasing despite the good therapeutic options for HA and HB, as about 10% of patients with severe hemophilia may experience severe (often fatal) perinatal hemorrhages [8].

In the present paper we discuss, also through clinical cases of an autosomal recessive model of disease (CF) or an X-linked model (HA and HB), some incidental findings that emerged from our work on PD that involve methodological, clinical, ethical, and legal aspects: (i) misattributed paternity; (ii) complex alleles; (iii) the incidental finding of disease in a parent; and (iv) the effect of de novo mutations.

## 2. Materials and Methods 

In this retrospective observational study, we included all PD for inherited genetic diseases performed in our Medical School from January 1993 to December 2018. Among the 1502 prenatal diagnoses (PDs) performed, we revised all cases of PD for CF (*n* = 239) as an example of autosomal recessive disease and for HA/HB (*n* = 47) as examples of X-linked diseases. All subjects gave their informed consent for inclusion before they participated in the study. The study was conducted in accordance with the Declaration of Helsinki.

All the procedures for PD of genetic inherited diseases have been detailed [2,3,4]. All the couples that received PD underwent a pre-test genetic counseling by a multidisciplinary team that included a physician with specific expertise in the disease, an obstetrician, a clinical molecular biologist, and, in some cases, a psychologist [9]. During the counseling it was detailed, among other topics, that PD may disclose misattributed paternity. After the counseling, women requiring PD underwent an ultrasound examination to assess the vitality of the embryo, placental site, and the pregnancy to date. 

The *CFTR* genotype was defined firstly by screening the most frequent *CFTR* gene mutations [10] and rearrangements [11]. Moreover, Sanger sequencing [12] or next generation sequencing [13] were used when mutations were not detected in one or both alleles by first-level analysis. Furthermore, we analyzed seven intragenic *CFTR* short tandem repeats [14] to perform PD of CF if one or both mutations were not known.

The analysis of *F8C* gene mutations was performed by long-PCR to test for IVS22 inversion and by two PCRs for IVS1 inversion analysis and, thus, by gene sequencing in cases negative to both inversions [5]. The analysis of *F9* gene mutations was performed by D-HPLC [15] or by direct gene sequencing [7].

## 3. Results

### 3.1. Misdiagnosis of CF

**Figure 1:**
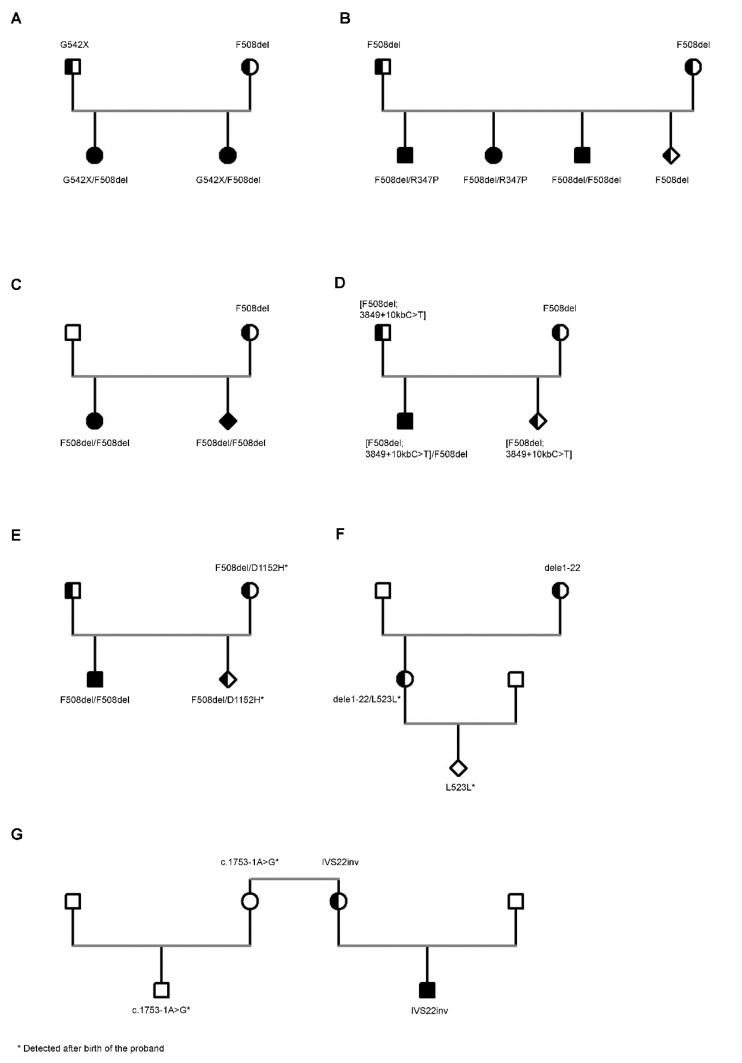
Clinical cases showing incidental findings related to prenatal diagnosis (PD) for cystic fibrosis (CF) and hemophilia A (HA). Misdiagnosis of CF (**A**). Misattributed paternity during CF molecular analysis (**B**,**C**). Complex alleles during PD for CF (**D**). Incidental diagnosis of CFTR-Related Disorders in a parent (**E**). The effects of de novo mutations in HA molecular analysis (**F**,**G**). Symbols: Square, male; circle, female; diamond, pregnancy, gender not specified; clear shape, unaffected subject; black shape, affected subject; shape shaded in half, carrier subject.

Case 1 (Figure 1A): This case describes two subsequent PDs for CF performed in another laboratory in the same family, in which both the parents had been defined as CF carriers based on family history. The first PD revealed the fetus as carrier of the F508del mutation. A female child was born, and the newborn screening (NBS) for CF was not performed because at that time it was not available in all newborns in our region. About one year later, the second PD was performed in the same laboratory, and again the fetus was revealed as a carrier of the F508del mutation. The second female child was born, and again NBS was not performed. One year later, both the children were referred to our laboratory, suspected of being clinically affected by CF; both revealed an altered sweat test (sweat chloride: 96 and 98 mmol/L in the first and 104 and 95 mmol/L in the second). Molecular analysis revealed the G542X/F508del genotype in both the children and confirmed the father as carrier of the G542X and the mother as carrier of the F508del mutation. The reports of both PDs resulted negative for the G542X mutation.

### 3.2. Misattributed Paternity

Case 2 (Figure 1B): This case describes the PD for CF in a couple that had a child affected by CF with the F508del/F508del genotype and two other older siblings not clinically affected by CF, with normal sweat test in both the cases, in which molecular analysis had not been performed. We performed PD at the 11th week of gestation testing DNA from CV. We excluded the contamination of fetal DNA by maternal DNA. Thus, we performed the analysis of parental mutations on DNA from CV that revealed the F508del heterozygous mutation. Both the parents were carriers of the mutation. Some years later the older siblings asked to be tested for their carrier status in another laboratory, without knowing the results of the molecular analysis of the other members of the family; thus, they were tested for a panel of the most frequent mutations. Surprisingly, both of the results were compound heterozygous for the F508del and R347P mutations. One of the two siblings, male, currently aged 21 years old, was revealed as being affected by congenital bilateral absence of vasa deferentia (CBAVD) with no other symptoms of CF after a careful clinical evaluation. The sweat test result was borderline (sweat chloride: 45 and 42 mmol/L). The other sibling, female, currently aged 28 years old, was revealed to be affected by diffuse bronchiectasis in the absence of other signs or symptoms of CF/CFTR-related disorders (RD). The sweat test was in turn borderline (i.e., 50 and 46 mmol/L). Both the siblings were referred to our laboratory to again perform molecular analysis that confirmed the F508del/R347P genotype in both. The R347P had not been identified in the parents or in the two other siblings, all tested with a panel including the mutation.

Case 3 (Figure 1C): This case describes the PD for CF in a couple that had a child affected by CF with the F508del homozygous genotype. We performed PD at the 11th week of gestation testing DNA from CV. We excluded the contamination of fetal DNA by maternal DNA. Molecular analysis revealed the mother as carrier of F508del, while the father was negative to F508del and to a panel of the most frequent *CFTR* mutations. We excluded large deletions that may mask F508del. Thus, we performed the analysis of F508del on DNA from CV that revealed the F508del homozygous mutation. The parents were counseled about the result of CF PD, and the mother opted for pregnancy termination. During post-test counseling, the finding of misattributed paternity was not discussed because the parents had asked to not be informed during the pre-test counseling. Later, the mother asked to be counseled alone, and we suggested that she inform the biological father of the fetus of his carrier status.

Among our 239 PDs for CF, we found four cases of misattributed paternity (1.7%). Three were previously described, and another is shown in the present study (Case 3).

### 3.3. Complex Alleles

Case 4 (Figure 1D): This case describes the PD for CF in a couple that had already a child affected by CF with the F508del/F508del genotype. Both the parents were tested for the F508del mutation, and results showed that both were carriers. We performed PD at the 11th week of gestation testing DNA from CV. We excluded the contamination of fetal DNA by maternal DNA. Thus, we analyzed DNA from CV using a panel of the most frequent mutations that revealed the F508del and the 3849 + 10 kb C > T mutations, both heterozygous. Thus, we tested again both the parents for 3849 + 10 kb C > T, and the results for the father were heterozygous for this mutation. We tested the affected child for the 3849 + 10 kb C > T and for the F508del mutations, and his results were homozygous for the F508del and heterozygous for the 3849 + 10 kb C > T. Thus, the 3849 + 10 kb C > T was in cis with the F508del, giving rise to a complex allele.

### 3.4. Incidental Diagnosis of CF/CFTR-RD

Case 5 (Figure 1E): This case describes the PD for CF in a couple that had already a child affected by CF with the F508del/F508del genotype. The analysis of the F508del mutation revealed both the parents as carriers. We performed PD at the 11th week of gestation testing DNA from CV. We excluded the contamination of fetal DNA by maternal DNA. Thus, we analyzed parental mutations on DNA from CV that revealed the F508del heterozygous genotype. Following a normal delivery, the NBS results were positive. Thus, we performed *CFTR* gene sequencing that revealed the F508del/D1152H genotype; the sweat test was positive twice (sweat chloride: 89 and 94 mmol/L), and the patient was diagnosed with CF. Molecular analysis performed on the parents by whole-gene sequencing revealed the mother as F508del/D1152H; the D1152H was excluded in the CF-affected sibling (thus excluding that it was in cis with the F508del). The mother was asymptomatic, the sweat test was borderline (sweat chloride: 44 and 52 mmol/L), and a careful clinical evaluation excluded any sign or symptom suggestive of CF or CFTR-RD. The anamnesis revealed some episodes suggestive of acute pancreatitis (recurrent pancreatitis); the instrumental (ultrasound and computed tomography scanning) and laboratory evaluation of the pancreas (including steatocrit and fecal elastase) were normal. She was diagnosed with CFTR-RD.

Following two cases in which we incidentally identified a second mild mutation in subjects that carried a severe CF mutation (mostly parents of patients with CF), we planned, either for new cases of CF and for PD, to scan the whole *CFTR* gene in the parents. So far, among 150 cases studied, we have found a second mild mutation (in addition to that transmitted to the affected son) in eight subjects (5.3%). All these cases were referred to the Adult Regional Reference Centre for CF, and seven out of eight were diagnosed with CFTR-RD for borderline sweat test and recurrent pancreatitis (three cases), diffuse bronchiectasis (three cases), and distal intestine obstructive syndrome (one case).

### 3.5. De Novo Mutations

Case 6 (Figure 1F): This case describes a PD for HA. It was performed on CV from a pregnant woman that was a known carrier of HA, being a cousin of an HA patient, and she was positive for the family mutation (a large deletion involving exons 1 to 22). The PD on CV sampled at the 11th week of gestation was negative for the large deletion. The child was born with natural delivery, and at the age of 10 years, he started to show hemorrhages for mild trauma. The analysis of FVIII revealed an activity of 6.7% (corresponding to a mild HA). We performed the whole scanning of *F8C* gene and identified the L523L mutation. The same mutation was identified in the mother that, thus, had the dele1-22/L523L genotype, but she was completely asymptomatic. The grandmother was negative to the L523L mutation, being a carrier only of the dele1-22 mutation, and the grandfather resulted negative to the analysis of the L523L mutation, suggesting that the mutation originated as a de novo mutation in the mother of the proband [16]. 

Among 47 PDs for HA/HB, this is the lone case in which a de novo mutation was demonstrated. However, we described another case [16] in which a de novo mutation of *F8C* gene was found in a male patient born with no PD because the family mutation (IVS22 inversion) had been excluded in the mother (Figure 1G).

## 4. Discussion

The cases presented in this paper show some incidental findings related to PD of inherited genetic diseases that raise methodological, clinical, ethical, and legal implications. Case 1 (that occurred about 15 years ago), Figure 1A, described the effects of a laboratory mistake. During the first PD, the laboratory missed the identification of the G542X mutation in the fetus (despite the fact that it was among the mutations tested), reporting that the fetus was positive only to the F508del mutation. During the second PD (one year later), again the laboratory did not identify the G542X mutation (again among the mutations tested) and also reported the second fetus as a carrier of the F508del mutation alone. The mistake clearly is due to a systematic technical error that caused the missed identification of the G542X twice; the analysis of the parents (one of which was a known carrier of the G542X mutation) probably would have avoided performing two consecutive errors.

Cases 2 and 3 (Figure 1B,C) raise the problem of the incidental finding of misattributed paternity, a situation that is becoming even more frequent as a consequence of the spread of genetic testing. The real frequency of misattributed paternity is unknown. Older studies reported a rate of 10% in the general population, while a recent survey of 67 studies reported a frequency of about 1.9% [17], not far from that obtained in the present study of 1.7%. In most cases, the incidental finding of misattributed paternity does not have medical consequences or a decision-making impact, as in Case 2 because the two patients diagnosed as F508del/R347P were adult and independent of the parents, and the identification of misattributed paternity does not change their reproductive risk (or that of the parents that are carriers of CF). In Case 3, however, the misattributed paternity impacts on the couple, modifying their reproductive risk and impacting the natural father, who is the unaware carrier of a severe mutation. Thus, he has a risk of 1:25 (frequency of CF carriers in the general population) that the partner is a CF carrier in turn and a risk of 1:100 to generate a son with CF. These cases raise relevant questions to healthcare professionals on (i) whether misattributed paternity must be disclosed, (ii) who should provide the communication and how, and (iii) which family members must receive information and in what order. There is no formal consensus between healthcare professionals, ethical committees, and legislation of different countries on how to handle the incidental discovery of misattributed paternity [18]. For example, federal law in the USA states that patients (or their guardians) have a right to all test results, and the President’s Commission for the Study of Ethical Problems in Biomedical and Behavioral Research in the States indicates that when both parents undergo genetic counseling, they are equal clients and, therefore, the counselor has a moral obligation to disclose the test results to both [18]. Some studies suggest the mother should be told about non-paternity before the presumed father to respect her privacy, then the man should be informed by the woman during counseling or in a group context with social support [19].

A survey of genetic counselors [20] and medical geneticists [21] found that more than 95% of them would not disclose to the father the misattributed paternity, and most of them suggested they would opt to tell the mother in private. This was our conduct in Case 3, also taking into account the relevance of suggesting the mother reveal the carrier status to the natural father of the fetus.

Recent declarations of the Italian courts stated that “the information given by the physician (counselors) to the patient has to be complete”, and the Italian Personal Data Protection Code states that “after receiving complete information about all aspects of PD during the counseling, the couple must decide whether or not to have the paternity disclosed to them”, so the problem will no longer remain that of the counselor [22]. According to these recent indications, we added in the informed consent before PD and in the pre-test counseling the clear indication that the test would incidentally reveal misattributed paternity, and we asked the couples to select between three options: (i) to not be informed of misattributed paternity, (ii) to be informed of misattributed paternity, and (iii) to be informed only if such data may have decisional implications (i.e., a different risk for the couple to generate an affected son).

A second incidental finding is that of the complex alleles (i.e., a second mutation in cis with another mutation previously identified). It is important to define that the two mutations are in cis because the complex allele means that the proband is a carrier (Case 4, Figure 1D), while two heterozygous mutations in trans mean that the proband is affected (see Case 5, Figure 1E). In CF, there is about a dozen frequent complex alleles [23]; thus, when a mutation included in such group is identified, it is reasonable to test for the other mutations that form the complex allele. However, it is possible that novel, complex alleles between *CFTR* mutations are identified [24], as occurred in our Case 4; thus, according to European guidelines [25] the analysis of the parents is mandatory even if the proband results are compound heterozygous for two mutations to define their allele status. However, the incidental identification of a second (usually not severe) *CFTR* mutation in trans with a severe mutation on the other allele may led to the diagnosis of CF in a subject previously considered as carrier. In the subjects described in the present study (Case 5), the second mutation (i.e., the D1152H) was identified in a newborn child diagnosed as a carrier of the severe F508del mutation because he resulted positive at the NBS. The mutation was also present in the mother, previously defined as a carrier of the F508del, and asymptomatic. Until some years ago, molecular diagnosis of CF was based on a panel of the most frequent *CFTR* mutations [10], and once two mutations in a patient diagnosed with CF were identified, such “familial” mutations were tested in blood relative subjects to define their carrier status and in PD if the couple planned another pregnancy. With the spread of gene-scanning techniques, the identification of further mutations in subjects previously defined as a carrier increased. Of course, the identification of a second, mild mutation makes it very difficult to define the clinical status of the patient. The D1152H is just an example of this situation for the large variability of its clinical impact [26]. In Case 5, the young patient was positive for NBS, he had altered sweat test, and thus he was diagnosed with CF. The mother had so far been asymptomatic, but after the analysis she was revealed as CFTR-RD. The same occurred for a series of other mild *CFTR* mutations that we found in patients with CFTR-RD [12]. Considering that in our small experience the incidental finding of a second mild mutation in subjects who are carriers of a severe *CFTR* mutation is not so rare (i.e., about 5% of subjects), and that most recent guidelines underlie the importance to also follow up patients with CFTR-RD to prevent complications, the question that is raised from these results is whether in a carrier of a severe *CFTR* mutation it would be useful to scan the whole gene, looking for a possible second mutation.

Finally, de novo mutations occur with high frequency in X-linked diseases because chromosome X, differently from autosomes, is more susceptible to mutational events [5]. The occurrence of novel mutations in families with HA or HB is well known in the context of molecular diagnostics. In fact, about a half of hemophilic patients appear as a novel case because of a new mutational event in the family, with no family history of the disease, and molecular analysis in such cases is based on the whole scanning of *F8C* or *F9* genes [5]. De novo mutations may impact also on PD, as is shown by cases 6 and 7 (Figure 1F,G). In both the cases, once excluding the family mutation, the residual risk for hemophilia was similar to that observed in the general population, and such risk is probably too low to perform the scanning of the whole gene looking for a possible de novo mutation. Of course, this point must be well known to the staff that perform pre-test counseling and must be clarified to the couples that ask for PD.

## 5. Conclusions

The increase of PD for inherited genetic diseases may cause a series of incidental findings that impact both the protocols and the counseling of the patients. Our data strongly suggest some points: (i) during PD it is mandatory to study the parents and to avoid errors and misinterpretation; (ii) the classic approach to CF of studying the proband and, thus, searching the two proband mutations in the parents is declining, thanks to the spread of gene-scanning techniques at a reasonable cost, which would suggest the need to study the whole *CFTR* gene both in the proband and in the parents; and (iii) there is a need to have international guidelines on the counseling of the patients, specifically focused on all the complexity that genetic analysis may propose.

In the meantime, it is necessary to carefully counsel the families, exploring and discussing all the different aspects, including incidental findings, and this task can only be performed by a multidisciplinary team that includes all professionals involved in PD. Of course, during the counseling, the family would be free to ask for all the results that they wish to be informed of.

## References

[1] Breveglieri G., D’Aversa E., Finotti A., Borgatti M. (2019). Non-invasive Prenatal Testing Using Fetal DNA. Mol. Diagn. Ther..

[2] Maruotti G.M., Frisso G., Calcagno G., Fortunato G., Castaldo G., Martinelli P., Sacchetti L., Salvatore F. (2013). Prenatal diagnosis of inherited diseases: 20 years’ experience of an Italian Regional Reference Centre. Clin. Chem. Lab. Med..

[3] Tomaiuolo R., Nardiello P., Martinelli P., Sacchetti L., Salvatore F., Castaldo G. (2013). Prenatal diagnosis of cystic fibrosis: An experience of 181 cases. Clin. Chem. Lab. Med..

[4] Terlizzi V., Lucarelli M., Salvatore D., Angioni A., Bisogno A., Braggion C., Buzzetti R., Carnovale V., Casciaro R., Castaldo G. (2018). Clinical expression of cystic fibrosis in a large cohort of Italian siblings. BMC Pulm. Med..

[5] Castaldo G., D’Argenio V., Nardiello P., Zarrilli F., Sanna V., Rocino A., Coppola A., Di Minno G., Salvatore F. (2007). Haemophilia A: Molecular insights. Clin. Chem. Lab. Med..

[6] Santacroce R., Acquila M., Belvini D., Castaldo G., Garagiola I., Giacomelli S.H., Lombardi A.M., Minuti B., Riccardi F., Salviato R. (2008). Identification of 217 unreported mutations in the F8 gene in a group of 1,410 unselected Italian patients with hemophilia A. J. Hum. Genet..

[7] Belvini D., Salviato R., Radossi P., Pierobon F., Mori P., Castaldo G., Tagariello G. (2005). Molecular genotyping of the Italian cohort of patients with hemophilia B. Haematologica.

[8] Zarrilli F., Sanna V., Ingino R., Santamaria R., Rocino A., Coppola A., Di Minno G., Castaldo G. (2013). Prenatal diagnosis of haemophilia: Our experience of 44 cases. Clin. Chem. Lab. Med..

[9] Maruotti G.M., Sarno L., Simioli S., Castaldo G., Martinelli P. (2013). Prenatal screening and counseling for genetic disorders. J. Matern. Fetal. Neonatal. Med..

[10] Tomaiuolo R., Spina M., Castaldo G. (2003). Molecular diagnosis of cystic fibrosis: Comparison of four analytical procedures. Clin. Chem. Lab. Med..

[11] Tomaiuolo R., Sangiuolo F., Bombieri C., Bonizzato A., Cardillo G., Raia V., D’Apice M.R., Bettin M.D., Pignatti P.F., Castaldo G. (2008). Epidemiology and a novel procedure for large scale analysis of CFTR rearrangements in classic and atypical CF patients: A multicentric Italian study. J. Cyst. Fibros..

[12] Amato F., Bellia C., Cardillo G., Castaldo G., Ciaccio M., Elce A., Lembo F., Tomaiuolo R. (2012). Extensive molecular analysis of patients bearing CFTR-related disorders. J. Mol. Diagn..

[13] Bergougnoux A., D’Argenio V., Sollfrank S., Verneau F., Telese A., Postiglione I., Lackner K.J., Claustres M., Castaldo G., Rossman H. (2018). Multicenter validation study for the certification of a CFTR gene scanning method using next generation sequencing technology. Clin. Chem. Lab. Med..

[14] Elce A., Boccia A., Cardillo G., Giordano S., Tomaiuolo R., Paolella G., Castaldo G. (2009). Three novel CFTR polymorphic repeats improve segregation analysis for cystic fibrosis. Clin. Chem..

[15] Castaldo G., Nardiello P., Bellitti F., Rocino A., Coppola A., di Minno G., Salvatore F. (2003). Denaturing HPLC procedure for factor IX gene scanning. Clin. Chem..

[16] Zarrilli F., Coppola A., Schiavulli M., Cimino E., Elce A., Rescigno G., Castaldo G., Amato F. (2018). Haemophilia A: The consequences of de novo mutations. Two case reports. Blood Transfus..

[17] Anderson K.G. (2006). How well does paternity confidence match actual paternity? Evidence from worldwide nonpaternity rates. Curr. Anthropol.

[18] Hercher L., Jamal L. (2016). An old problem in a new age: Revisiting the clinical dilemma of misattributed paternity. Appl. Transl. Genom..

[19] Mandava A., Millum J., Berkman B.E. (2015). When Should Genome Researchers Disclose Misattributed Parentage?. Hastings Cent. Rep..

[20] Pencarinha D.F., Bell N.K., Edwards J.G., Best R.G. (1992). Ethical issues in genetic counseling: A comparison of M.S. counselor and medical geneticist perspectives. J. Genet. Couns..

[21] Wertz D.C., Fletcher J.C. (1991). Privacy and disclosure in medical genetics examined in an ethics of care. Bioethics.

[22] Tozzo P., Caenazzo L., Parker M.J. (2014). Discovering misattributed paternity in genetic counselling: Different ethical perspectives in two countries. J. Med. Ethics.

[23] Terlizzi V., Castaldo G., Salvatore D., Lucarelli M., Raia V., Angioni A., Carnovale V., Cirilli N., Casciaro R., Colombo C. (2017). Genotype-phenotype correlation and functional studies in patients with cystic fibrosis bearing CFTR complex alleles. J. Med. Genet..

[24] Bergougnoux A., Boureau-Wirth A., Rouzier C., Altieri J.P., Verneau F., Larrieu L., Koenig M., Claustres M., Raynal C. (2016). A false positive newborn screening result due to a complex allele carrying two frequent CF-causing variants. J. Cyst. Fibros..

[25] Dequeker E., Stuhrmann M., Morris M.A., Casals T., Castellani C., Claustres M., Cuppens H., des Georges M., Ferec C., Macek M. (2009). Best practice guidelines for molecular genetic diagnosis of cystic fibrosis and CFTR-related disorders—Updated European recommendations. Eur. J. Hum. Genet..

[26] Terlizzi V., Carnovale V., Castaldo G., Castellani C., Cirilli N., Colombo C., Corti F., Cresta F., D’Adda A., Lucarelli M. (2015). Clinical expression of patients with the D1152H CFTR mutation. J. Cyst. Fibros..

